# Acute Kidney Injury associated with "Triple whammy" combination: a protocol for a systematic review.

**DOI:** 10.12688/f1000research.109987.1

**Published:** 2022-05-05

**Authors:** Dulce Maria Calvo Barbado, Luis Carlos Saiz Fernández, Leire Leache Alegría, Maria Concepción Celaya Lecea, Marta Gutiérrez-Valencia.

**Affiliations:** 1 PhD Program, Autonomus University of Barcelona, Barcelona, Barcelona, 08193, Spain; 2 Unit Innovation and Organization, Navarra Health Service, Pamplona, Navarra, 31004, Spain; 3 Pharmacy, Navarra Health Service, Pamplona, Navarra, 31004, Spain

**Keywords:** Acute kidney injury, triple whammy, diuretics, Angiotensin-Converting Enzyme Inhibitors, Angiotensin II Type 1 Receptor Blockers, Non-Steroidal Anti-Inflammatory Agents, metamizole, dipyrone

## Abstract

**Background:** “Triple whammy” (TW) refers to the simultaneous use of diuretics, renin-angiotensin-aldosterone system inhibitors and nonsteroidal anti-inflammatory drugs (NSAIDs). To date, the risk of developing acute kidney injury (AKI) associated to this combination has not been deeply investigated. The objectives are to analyze the incidence of AKI associated to the exposure to “triple whammy” including all NSAIDs versus non-exposure to this combination. Secondarily, the risk of hospitalization, severe adverse events, requirement of renal replacement therapy and mortality will be assessed. Also, the incidence of AKI associated to the exposure to “triple whammy” versus non-exposure will be analyzed, including only metamizole as NSAID.

**Methods:** A systematic literature search of intervention studies and analytical observational studies will be conducted in the Cochrane Library, Medline and EMBASE, among others. AKI 12 months after the last prescription of the triple combination will be the main outcome. Relative frequencies, risk of bias and certainty of evidence will be analyzed. Additionally, sensitivity and subgroup analyses will be performed.

**Results:** Once this systematic review has been completed, the results are expected to provide an estimate of the risk associated with this triple combination and the renal variables, in addition to new guidance on the renal treatment of patients potentially receiving triple therapy.

**Conclusions: **This is intended to be the first systematic review of observational studies to analyse TW combination and AKI's risk based on well-validated epidemiological databases exploring drug safety issues.

## List of abbreviations

AECI: Angiotensin-Converting Enzyme Inhibitors

AKI: Acute Kidney Injury

ARB: Angiotensin II Type 1 Receptor Blockers,

GRADE: Grading of Recommendations, Assessment, Development and Evaluation

KDIGO: Kidney Disease Improving Global Outcomes

NSAID: Non-steroidal anti-inflammatory drugs

ROBINS-I: Risk Of Bias In Non-randomized Studies of Interventions

WHO: World Health Organization

## Background

The term “triple whammy” corresponds to the simultaneous use of diuretics, antihypertensive inhibitors of the renin system angiotensin (Angiotensin-Converting Enzyme Inhibitors (ACEi) or Angiotensin II Type 1 Receptor Blockers (ARB) and non-steroidal anti-inflammatory drugs (NSAIDs). ^1^ These groups of drugs affect kidney function through different mechanisms such as producing hypovolemia, and reduction of glomerular filtration rate and glomerular perfusion. ^2^

This combination of drugs has been correlated with an increased incidence of Acute Kidney Injury (AKI). ^2^ In this sense, Loboz *et al.* found a significant association between the number of drugs components of the triple therapy and the deterioration of renal function, established as an increase of serum creatinine or reduction of creatinine clearance. ^3^ Specifically, the triple association has been associated with an increase of about 30% in the risk of AKI. ^4^^,^^5^ Furthermore, in the case-control study carried out by Lapi *et al.* it was concluded that the risk of developing AKI was higher during the first 30 days of treatment with the triple therapy. ^6^

AKI has important social and economic consequences. There is a wide variation in prevalence (1-25%) and mortality (15-60%) estimates for AKI due to the existence of multiple pathology definitions. ^7^ In economic terms, the cost associated with AKI is around 5% of the hospital budget and 1% of total health expenditure, due to an increase in hospital stay, intensified monitoring and dialysis requirements. ^8^^,^^9^

In Spain, there has been an exponential increase in the number of hospitalizations with diagnosis of AKI over time. ^10^ The number of hospitalizations for AKI in 1997 was 15,885, reaching 207,287 hospitalizations in 2015. ^10^ Iavecchia *et al.* found that 54.1% of the hospitalizations were related to drugs that were diuretics and ones that acted on the renin-angiotensin system. ^11^ García Camín *et al.* showed that the incidence of out-of-hospital AKI due to drugs considered in the “triple whammy” association was 3.4 cases per 1,000 consumers/year, with a mortality of 11.3% during hospital admission and 38.7% at 12 months. In addition, an average avoidable cost of 214,604 euros per 100,000 inhabitants/year was estimated. ^12^

Scientific evidence of the “triple whammy” combination effect from studies is scarce. The initial review of the literature provides three nested case control studies conducted in the United Kingdom, ^4^ Canada, ^6^ and the United States. ^13^ Descriptive, cross-sectional, and retrospective studies in Australia ^3^^,^^14^ and Japan. ^5^ Only three investigations carried out in Spain have been identified, ^12^^,^^15^^,^^16^ of which only two analyzed the incidence of AKI, mortality and costs associated with “triple whammy”. The third study analyzed the effect of pharmaceutical interventions on the frequency of prescription of the combination therapy.

Two Pharmacovigilance studies conducted in France have also provided information on this association and the risk of AKI. ^17^^,^^18^

In contrast with other countries, metamizole is used largely for pain management in Spain. Safety data from the WHO international database VigiAccess contains 23,582 reports of adverse reactions related to metamizole. Of these, 534 (2.3 %) affect the renal and urinary system, with 197 (37.6 %) corresponding to acute renal failure and 112 to related terms (21%), 57% reports related to metamizole come from Europe. ^17^ The European database of suspected adverse reactions ( Eudravigilance) contained 9,906 reports with metamizole up to June 2020, of which 25.2% come from Spain. In total, 5.3% of the reports corresponded to reactions affecting the renal and urinary system, of which 261 (49.4%) were recorded as acute renal failure, and 26 (4.9%) reported severe reactions. ^17^

Despite these reported data, there is no strong evidence showing that metamizole relates to a lower risk of AKI compared to other types of NSAIDs. To date, studies examining the effect of metamizole in combination with diuretics and ACE inhibitors/ARBs on the risk of developing AKI have also not been conducted. Therefore, it is particularly relevant to determine the actual impact of metamizole on kidney function.

Review question: Do patients exposed to the “triple whammy” combination with/without metamizole have a higher risk of AKI compared those not exposed to the “triple whammy” combination?

The main objective is to analyze the incidence of AKI associated to the exposure versus non-exposure of the “triple whammy” combination.

## Methods

This protocol has been registered with PROSPERO international prospective register of systematic reviews (registration number CRD42020213680) and has been reported in accordance with the Preferred Reporting Items for Systematic Reviews and Meta-Analysis Protocols (PRISMA-P) 2015 statement ( See Reporting Guidelines). ^18^

The methodology of this review is according to the Cochrane Handbook for Systematic Review of Interventions. ^19^

## Eligibility criteria

### Type of studies

Clinical trials and analytical observational studies assessing the risk of AKI or morbidity-mortality associated with the “triple whammy” combination will be included. Priority will be given to randomized controlled trials, but non-randomized controlled trials, controlled before-after studies, historically controlled studies, cohort studies and case-control studies will also be considered for inclusion. In order to be included, studies must have analyzed the primary outcome or at least one secondary outcome. There will be no restrictions regarding the publication date, setting and languages. Animal studies will be excluded.

### Type of participants

Patients ≥ 18 years will be included. Patients with any type of cancer, except from basal cell carcinoma, will be excluded.

### Exposure

Exposure to the “triple whammy” combination will be analyzed. The combination refers to the simultaneous use of at least one diuretic of any class, plus ACEI or ARB or aliskiren plus NSAIDs.

### Comparator/control

No exposure to the “triple whammy” combination.

### Outcome variables

Acute Kidney Injury (AKI) will be the main outcome. Secondary outcome variables will be the following: hospitalization due to AKI, serious adverse events, mortality within 30 days after AKI, mortality from any cause and requirement of kidney replacement therapy. Events (primary and secondary outcomes) within 12 months after the last prescription of the triple combination will be considered, except for mortality within 30 days after AKI.

### Condition or domain under study

The definitions of AKI used in the different studies will be accepted to determine the occurrence of AKI. In the case of studies that do not provide a certain AKI definition, the definition established by Kidney Disease Improving Global Outcomes (KDIGO) will be adopted.

According to the criteria established by KDIGO, AKI is defined as an increase in serum creatinine of 26 micromol/liter (0.3 mg/dl) or more within 48 hours, or a 50% or greater increase in serum creatinine known or suspected to have occurred in the last 7 days or a drop in urine output to less than 0.5 ml/kg/hour for more than 6 hours in adults, plus 25% of the estimated glomerular filtrate value in the last 7 days. ^20^^–^^23^

### Sources of information

A systematic search of the literature will be conducted through the following sources: Cochrane Library, Medline, Epistemonikos, EMBASE, Scopus, Web of Science, CINAHL, Latin American and Caribbean Health Sciences Literature (LILACS) Bireme and Scientific Electronic Library Online (Scielo).

Primary studies will be identified through the ClinicalTrials.gov database, the Spanish Clinical Trials Registry (REEC), the EU Clinical Trials Registry, the EU post-authorization study registry (ENCePP), and the International Clinical Trials Registry Platform (ICTRP) of the World Health Organization. A search in PROSPERO will be carried out to identify completed and/or ongoing reviews. In addition to the electronic database search, citation tracking (checking reference lists of all included and excluded studies) will be performed to search for other relevant articles and to identify additional trials and studies. References from reviews, clinical practice guidelines and position papers from health care institutions or organizations will be accessed.

### Search strategy

The strategy will be built with the following keywords and boolean operators for the initial question:

((“Angiotensin-Converting Enzyme Inhibitors” [Mesh]) OR (Angiotensin-Converting Enzyme Inhibitors [Pharmacological Action]) OR (Angiotensin Receptor Antagonists [Mesh]) OR (Angiotensin II Type 1 Receptor Blockers [Mesh])) AND ((Diuretics [Mesh]) OR (Diuretics [Pharmacological Action])) AND ((Anti-Inflammatory Agents, Non-Steroidal [Pharmacological Action]) OR (Anti-Inflammatory Agents, Non-Steroidal [Mesh]) OR (Dipyrone [Mesh]) OR (Metamizole magnesium [Supplementary Concept])) OR (“triple whammy”)) AND (“Acute Kidney Injury” [Mesh]).

### Screening and data extraction

Two independent reviewers will carry out the selection of papers based on the title, keywords and abstract, and eligibility criteria. The full text of each paper considered for inclusion will be obtained. If the information relevant to the inclusion criteria is not available in the abstract or if the title is relevant but the abstract is not available, the full text of the report will be obtained. Discrepancies will be resolved by discussion or by a third author if necessary. A PRISMA (Preferred Reporting Items for Systematic Reviews and Meta-Analyses' statement) flowchart of included and excluded articles will be prepared.

Two reviewers will independently extract the data from the included trials with a previously prepared data extraction form. Differences between authors will be resolved by consensus and the participation of a third author, if necessary. The data extraction form will include descriptive information of the included studies (general data, eligibility, inclusion and exclusion criteria, methodology used, and assessment of risk of bias) and information on primary and secondary outcomes. Microsoft Access and Microsoft Excel will be used to organize and analyze the data of individual participants. If it is deemed necessary to complete the information, the authors will be contacted for additional data. Whenever possible, analyses will be carried out using the individual data from the included studies.

### Outcomes and prioritization

Acute Kidney Injury (AKI) occurring 12 months after the last prescription of the triple combination, or “triple whammy” will be the main outcome.

### Secondary outcomes

1. Hospitalization for AKI
2. Serious adverse events: Any unfortunate medical event that may occur during treatment with a drug but does not necessarily have a causal relationship with that treatment. In this case, the coincidence in time occurs without any suspicion of a causal relationship. The serious event of those that cause death, threaten the life of the patient, produce permanent or substantial disability, require hospitalization or prolong the time of hospitalization, produce congenital anomalies or malignant processes.
3. Mortality within 30 days after Acute Kidney Injury: only deaths occurring within 30 days after the AKI that must have occurred in the year following the exposure to the triple whammy will be considered.
4. Mortality from any cause.
5. Kidney replacement.

### Risk of bias and quality of the evidence

Risk of bias will be evaluated for each study using for randomized clinical trials, the Cochrane Risk of Bias 2 (RoB 2) tool, according to the method described in the Cochrane Handbook for Systematic Reviews of Interventions version 6.0, ^19^ the ROBINS-I tool ^24^ and Newcastle-Otawa Scale. ^25^ The Grading of Recommendations, Assessment, Development and Evaluations (GRADE) methodology ^26^ will be applied to evaluate the quality of the evidence for the main and secondary outcomes. A funnel plot will be carried out in the case of having 10 or more studies for each of the variables, in order to evaluate the possible existence of publication bias.

### Data analysis

Data synthesis and analysis will be performed using ( RevMan, RRID:SCR_003581) version #5.4. Exposure to the “triple whammy” combination (regardless of whether the NSAID is metamizole or another drug) will be considered for the main analysis. The secondary analysis will consider exposure to the triple whammy combination in which the NSAID is metamizole.

Randomized and non-randomized clinical trials will be analyzed separately. In addition, non-randomized studies will be analyzed separately according to the specific study design.

Relative frequencies will be used to compare the incidence or the frequency of the event among the patients exposed and not exposed to the triple combination. The risk ratio (RR) or odds ratio (OR), with their corresponding 95% confidence interval, will be used. The Chi ^2^ and the I ^2^ statistics will be used to describe the heterogeneity. Indication of substantial heterogeneity will be I ^2^ >60%.

When substantial heterogeneity (I ^2^ value greater than 60%) is found, the pooled effect estimator will not be provided; in this case, a narrative description of the results will be provided. With respect to observational studies, if both unadjusted and adjusted effect estimates are reported, the adjusted effects will be preferred.

Qualitative results should be presented in a narrative form.

### Subgroup analyses

Subgroup analyses will be carried out for the following populations: patients over 75 years; patients with previous chronic renal failure; patients with cardiovascular disease.

### Sensitivity analyses

Sensitivity analyses will be performed excluding studies with high risk of bias and including only those studies that used KDIGO's definition of AKI.

## Discussion

Beyond the individual well-known renal action of diuretics, AECI, ARB and NSAID, the effect of the combination and its potential association with the risk of AKI still needs to be explored. On the other hand, there is not much evidence on the use of metamizole and its risk. This systematic review will provide updated evidence relative to the risk of AKI and “triple whammy”. As a result, the project is expected to provide a new strategy to decrease the risk of renal injury in patients potentially using this triple combination.

This is a protocol for a systematic review and any amendment will be reported by updating the submitted protocol at PROSPERO (an international and prospective register of systematic reviews) and will be reported in the final manuscript. There is a plan to disseminate all results by means of a subsequent publication, which will be part of the evidence to support a nested case-control study on triple whammy and AKI in Spain.

To the best of our knowledge, there is a lack of high-quality data on this topic, which makes this research particularly timely.

## Conclusion

AKI is a growing global disease that causes major morbidity and death and has significant resource consequences. The concurrent use of nephrotoxic medications raises the risk of AKI. Based on their pharmacological mechanism, simultaneous administration of diuretics, antihypertensives such as RASI, and NSAIDs or metamizole, known as “triple whammy”, may be linked to AKI. To our knowledge, this is the first systematic review of case-control studies to analyse the “triple whammy” combination and AKI's risk based on well-validated epidemiological databases exploring drug safety issues. The evidence generated by this study can be used as a basis for future studies in order to better understand the risk of AKI and triple therapy combinations. These future studies could explore other confounding factors such as dose, individual’s pharmacokinetics, existence of arteriosclerosis, salt consumption, other medicines, and genetic polymorphisms in enzymes.

## Data availability

### Underlying data

No data are associated with this article.

## Reporting guidelines

Open Science Framework: PRISMA-P checklist for ‘Acute Kidney Injury associated with “Triple whammy” combination: a protocol for a systematic review’.

Calvo Barbado, Dulce Maria (2022): PRISMA-P 2015 checklisthalffull20032022.docx. figshare. Journal contribution. https://doi.org/10.6084/m9.figshare.19501465.v1. ^18^

Data are available under the terms of the Creative Commons Zero “No rights reserved” data waiver (CC0 1.0 Public domain dedication).

## Author contributions

Dulce Maria Calvo: Formal analysis (lead) writing – original draft (lead); investigation (lead), methodology (equal); review and editing (equal); guarantor (lead). Luis Carlos Saiz: Formal analysis (supporting), investigation (supporting), methodology (equal); writing – original draft (supporting); review and editing (equal). Leire Leache Alegría: Methodology (equal); writing – original draft (supporting); review and editing (equal). Maria Concepción Celaya: Conceptualization (lead); methodology (equal); review and editing (equal). Marta Gutiérrez Valencia: Methodology (equal); writing – original draft (supporting); review and editing (equal).
