# Supplementary material for: Acute Kidney Injury associated with "Triple whammy" combination: a protocol for a systematic review
Source: F1000Res. 2022 Jul 26;11:496. Originally published 2022 May 5. [Version 2] doi: 10.12688/f1000research.109987.2 (PMC9889944; doi:10.12688/f1000research.109987.2)
Supplement: Peer review history

# Peer review: Reviewer response for version 2

**DOI:** 10.5256/f1000research.136059.r156948

**Published:** 2023-01-31

**Authors:** Cesar Loza Muñarriz

**Affiliations:** 1 Nephrology Unit, National Hospital Cayetano Heredia, Lima, Peru

**Summary**

 Acute Kidney Injury (AKI) is a public health problem worldwide, due to the high rate of morbidity and mortality in the affected population. Concomitant use of nephrotoxic drugs increases the risk of AKI. The term "triple whammy" corresponds to the simultaneous use of diuretics, antihypertensive inhibitors of the renin angiotensin system (Angiotensin-Converting Enzyme Inhibitors (ACEi) or Angiotensin II Type 1 Receptor Blockers (ARB) and non-steroidal anti-inflammatory drugs (NSAIDs) or metamizole, which may be related to AKI. Scientific evidence of the effects of the "triple whammy" combination in studies is scant. But there are reports in the population of incidence, in-hospital mortality, and mortality at 1-year follow-up, and although relatively low, there is a great impact on costs.

**Suggestions**

1. If there are good-quality RCTs that comprehensively answer the question of interest, it is not recommended to include non-randomized studies, since there is a greater risk of bias.
2. If there are no high-quality RCTs and it is not expected that they will exist in the short term or it is not possible to perform them for ethical reasons or there are issues not resolved by the RCTs, and the answer to the research question is a priority for clinical decision-making, the inclusion of non-randomized studies should be considered, always seeking to ensure that the studies that are included have the least possible potential for bias.
3. It is important to detail in the protocol when evaluating non-randomized studies how investigators controlled for confounding factors; whether investigators controlled for confounding from design (for example, did they match using propensity ratios) or did they adjust for confounding in the analysis phase, and if so, should have defined the method used (eg stratification, multivariate regression or, again, propensity ratios used).
4. If researchers decide to present information from randomized clinical trials and non-randomized studies in the same Systematic Review, it is recommended to analyze them independently and comment on the similarities and differences found in the results, and perform a sensitivity analysis.

Is the study design appropriate for the research question?

Yes

Is the rationale for, and objectives of, the study clearly described?

Yes

Are sufficient details of the methods provided to allow replication by others?

Partly

Are the datasets clearly presented in a useable and accessible format?

Not applicable

Reviewer Expertise:

Nephrology and clinical epidemiology

I confirm that I have read this submission and believe that I have an appropriate level of expertise to confirm that it is of an acceptable scientific standard.

# Peer review: Reviewer response for version 1

**DOI:** 10.5256/f1000research.121551.r142589

**Published:** 2022-07-21

**Authors:** Antònia Agustí Escasany

**Affiliations:** 1 Department of Pharmacology, Therapeutics and Toxicology, Universitat Autònoma de Barcelona, Barcelona, Spain; 2 Clinical Pharmacology Service, Vall d'Hebron University Hospital, Barcelona, Spain; 3 Clinical pharmacology research group, Vall d'Hebron Institute of research, Barcelona, Spain

Acute Kidney Injury associated with "Triple whammy" combination: a protocol for a systematic review.

 Calvo Barbado DM, Saiz Fernández LC, Leache Alegría L, Celaya Lecea MC and Gutiérrez-Valencia. M

 The authors will perform a systematic review to calculate the incidence of AKI (Acute Kidney Injury) associated with triple whammy treatment apart from other secondary objectives. In the review clinical trials and also observational studies are going to to be included without any restriction regarding the publication date, setting and languages. The methodology of the review is correct and the authors provide with sufficient details in order to be able to replicate the results by others.

 The only comments I have are the following:

1. I do not know why the authors have chosen 12 twelve months as the period to consider the main and the secondary objectives of the study.
2. I agree to include in the review the different kind of design studies (clinical trials and the observations ones) and analysed them according to the design of the studies, but I think it could be quite a lot of work.

Is the study design appropriate for the research question?

Yes

Is the rationale for, and objectives of, the study clearly described?

Yes

Are sufficient details of the methods provided to allow replication by others?

Yes

Are the datasets clearly presented in a useable and accessible format?

Not applicable

Reviewer Expertise:

Pharmacovigilance and pharmacoepidemiology.

I confirm that I have read this submission and believe that I have an appropriate level of expertise to confirm that it is of an acceptable scientific standard.

## References

[1] : Acute Kidney Injury associated with. *F1000Research* .2022;11: 10.12688/f1000research.109987.1 10.12688/f1000research.109987.1 35464048

# Response

**Published:** 2022-07-24

**Authors:** Dulce Maria Calvo Barbado

**Affiliations:** Autonomus University of Barcelona, Spain

Dear Reviewer, Thanks for your comments and review process.

 Regarding your comments, maybe it is something that we will need to clarify in our protocol. We refer "Events (primary and secondary outcomes) within 12 months after the last prescription of the triple combination will be considered", due to AKI is not to be measured "at" 12 months but "in" the 12 months following the end of exposure to TW.  We sought to extend the AKIs not only to the time patients are exposed, but also to the following 12 months (beyond 12 months we interpret the AKI as not being due to previous exposure). Maybe we need to clarify more in our protocol to be understand better for readers.

 Regarding your second comments, we agree that it will be a lot of work, but thinking that it is a safety problem, observational studies can be a proper way to do it.

 Again we are thankful for your comments and will take in mind.

 Dulce Maria Calvo
